# Tickborne Encephalitis in Naturally Exposed Monkey (*Macaca sylvanus)*

**DOI:** 10.3201/eid1306.061173

**Published:** 2007-06

**Authors:** Jochen Süss, Ellen Gelpi, Christine Klaus, Audrey Bagon, Elisabeth M. Liebler-Tenorio, Herbert Budka, Bernhard Stark, Werner Müller, Helmut Hotzel

**Affiliations:** * Friedrich-Loeffler-Institute, Jena, Germany; † Medical University of Vienna, Austria; ‡ Affenberg Salem, Bodensee, Germany; § Labor ALOMED, Radolfzell, Germany

**Keywords:** tickborne encephalitis, monkey, natural infection, Macaca, flavivirus, tickborne encephalitis virus, dispatch

## Abstract

We describe tickborne encephalitis (TBE) in a monkey (*Macaca sylvanus*) after natural exposure in an area at risk for TBE. TBE virus was present in the brain and could be identified as closely related to the European subtype, strain Neudoerfl.

Tickborne encephalitis (TBE) is a zoonotic disease caused by TBE virus (TBEV), a flavivirus. There are 3 subtypes of TBEV, the European subtype, transmitted by the hard tick *Ixodes ricinus*, and the Far Eastern and Siberian subtypes. Except in Austria, where mass vaccination campaigns were organized, the incidence of TBE in humans has increased in the past 30 years in all European countries with regions with risk for the disease. The average increase of TBE in 10 European countries was 311% from 1974–1983 to 1994–2003 (*1*,*2*). In Germany TBE incidence has increased enormously during recent years.

Most TBE group viruses use rodents as maintenance and amplifying hosts. TBE is predominately reported in humans, seldom in dogs and horses, and is as yet unknown in monkeys.The clinical progress of TBE is typically biphasic. In humans, a nonspecific influenzalike illness develops as the first phase of illness 7-14 days after they are bitten by a TBEV-positive tick. A second phase, with central nervous system involvement, develops in ≈30% of patients. Initial signs and symptoms include meningitis, encephalitis, and radiculitis. The case-fatality rate is 1%–2% in central Europe and 20%–40% in Siberia and the Far East.

## The Study

On July 14, 2006, staggering paresis of the hind legs, incoordination, and intermittent opisthotonos developed in a female barbary macaque (*Macaca sylvanus*). No nystagmus was present. The monkey, born April 27, 2005, was from a group of ≈200 animals living in 3 social groups. The animals were kept in a large, outdoor enclosure of a monkey park situated in a TBE-risk area in southern Germany. Four days after the onset of clinical signs, the animal became comatose and was euthanized. Blood was collected before euthanasia, and serum was prepared.

At necropsy, no macroscopic lesions were observed. The brain was removed and immediately frozen on dry ice and sectioned in 2-cm slices. Alternating slices were placed in 3.5% neutral buffered formalin at 4°C. After 48 h, representative areas, including cerebral cortex, hippocampus, basal ganglia, and cerebellum, were embedded in paraffin. Sections (3–5 μm) were cut from each block and stained with hematoxylin and eosin for standard histopathologic evaluation. For immunohistochemical detection of TBEV antigens, a noncommercial rabbit polyclonal hyperimmune serum (dilution 1:1,000) was used as described previously (*3*).

Histologic examination of the brain tissue could be satisfactorily performed despite moderate artifacts caused by freezing (Figure, Panel A). Moderate perivascular inflammatory cuffs and slight diffuse infiltration of brain parenchyma by mononuclear cells were observed in almost all brain areas, including basal ganglia and cerebellum (Figure, Panel B). In addition, slight mononuclear inflammatory infiltrates were present in the meninges. Microglial nodules were not detected. Immunohistochemical testing demonstrated several anti-TBEV immunoreactive neurons and processes, mainly in Purkinje cells of the cerebellar cortex (Figure, Panel B), and to a lesser extent in pyramidal neurons of the temporal cortex. Single neuronophagias were also observed.

**Figure:**
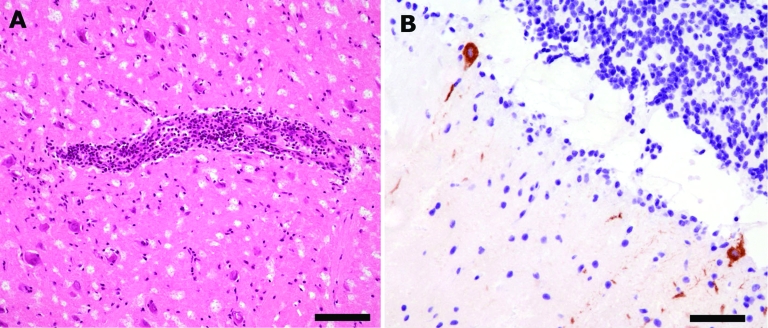
A) Moderate perivascular inflammatory infiltrates and slight diffuse infiltration of brain parenchyma by mononuclear cells in basal ganglia (hematoxylin and eosin stain, bar = 110 μm). B) Immunohistochemical findings for tickborne encephalitis virus (TBEV): strong immunolabeling of cerebellar Purkinje cell perikaryon and apical dendrites (anti-TBEV, bar = 60 μm).

From the frozen material, 10 samples of brain tissue were selected for PCR analysis, including cerebrum, cerebellum, and brain stem (Table). Viral RNA was extracted from brain tissue with RNeasy Kit and from cerebrospinal fluid with QIAamp Viral Kit (both from QIAGEN, Hilden, Germany). The brain tissue was homogenized by bead-milling (Retsch, Haan, Germany) with 3-mm stainless steel beads in 0.5 mL of lysis buffer, and the QIAshredder system (QIAGEN) was used to improve homogenization. A modified nested reverse transcription–PCR (nRT-PCR) was conducted with primer pairs Pp1, Pm1 (reverse transcription and first PCR), and Pp2, Pm2 (nested PCR) (*4*). Amplification was done in a 50-μL reaction volume containing 10 μL 5× buffer, 2 μL deoxynucleotide triphosphate mix (10 mmol/L), 2 μL enzyme mix (all from QIAGEN), 2 μL RNase inhibitor (Promega, Mannheim, Germany), 1.25 μL primers Pp1 and Pm1 (20 pmol/μL; Jena-Bioscience, Jena, Germany), 27.5 μL diethyl pyrocarbonate (DEPC)–treated water, and 4 μL RNA extract. The reaction was performed in an Eppendorf (Hamburg, Germany) thermal cycler for 30 min at 60°C for RT and 15 min at 95°C for denaturation as the initial step, followed by 40 cycles of PCR with 30 s at 94°C, 30 s at 66°C, and 1 min at 72°C. Final extension was 10 min at 72°C.

**Table:** Results of nested reverse transcription–PCR (nRT-PCR) analysis of the tickborne encephalitis virus (TBEV)–infected macaque brain

Sample no.	Specimen	TBEV results by nRT-PCR*	TBEV strain (sequencing)
06F0927			
T653	Cerebellum	+	Neudoerfl†
T654	Cerebellum	–	–
T655	Neocortex	+	Neudoerfl†
T656	Neocortex	–	–
T659	Neocortex	+	Neudoerfl†
T660	Neocortex	–	–
T657	Brain stem	+	Neudoerfl†
T658	Brain stem	–	–
T661	Brain stem	+	Neudoerfl
T662	Brain stem	+	Neudoerfl†
06F0926			
T663	Cerebrospinal fluid	–	–
Total		6/11	

*+, virus detected; –, no virus detected. †Differs by 1 nt from strain Neudoerfl, the prototype strain of the European virus subtype.

The second amplification reaction was carried out with 4 μL of amplification product in a 50-μL reaction (25 μL *Taq* PCR Master Mix [QIAGEN], 1.25 μL of each primer Pp2 and Pm2 [20 pmol/μL], and 18.5 μL DEPC-treated water). After a denaturation step of 2 min at 95°C, 30 cycles of 30 s at 94°C and 30 s at 65°C were performed, followed by 10 min at 72°C.

PCR products (178 bp) were visualized under UV light after electrophoresis on 1.5% agarose gel and ethidium bromide staining. Bands were cut out, and DNA was extracted by using the QIAquick Gel Extraction Kit (QIAGEN). DNA sequencing was conducted by cycle sequencing, using the BigDye Terminator Cycle Sequencing Kit (Applied Biosystems, Darmstadt, Germany) according to the manufacturer’s instructions. Amplification primers Pp2 and Pm2 were also used as sequencing primers. Nucleotide sequences were determined on an ABI Prism 310 Genetic Analyzer (Applied Biosystems).

Six of the10 brain samples were positive and CSF was negative for TBEV by nRT-PCR (Table). The sequences of these 6 PCR products (178 bp) differed by only 1 nt from that of strain Neudoerfl, the prototype strain of the European virus subtype.

The serum was tested for whole specific TBE antibodies (immunoglobulin [Ig] G and IgM) by ELISA (*5*). Solid-phase bound antigen and antigen conjugate were from a commercially available test kit (Immunozym FSME, Progen, Heidelberg, Germany). As standards, negative and positive test serum samples were used, and the following limit values were defined: <5 U/L, negative; 5–7 U/L, border line; 9–14 U/L, weakly positive; >14 U/L, positive; and >50 U/L, strongly positive. The serum of the macaque described in this paper was positive for specific TBE antibodies (24 U/L)

## Conclusions

Experimental infections of macaques with TBEV and related flaviviruses (Kyasanur Forest disease virus, Powassan virus) have been reported (*6*–*8*), but natural infections with TBE virus have not been reported previously. In our case, clinical signs, neuropathologic findings, and immunohistochemical detection of TBEV antigen in neurons and of TBEV by nRT-PCR indicate that the macaque succumbed to natural TBEV infection. Although the classical multinodular pattern of lesions in the brain was not observed, the distribution of viral antigens was comparable to that observed in fatal human TBEV infection with a short clinical course. The TBEV was characterized as closely related to the European prototype strain Neudoerfl, which suggests that the infection was acquired locally by infected ticks. This fact is surprising because macaques generally quickly remove ticks during social grooming.

The monkey park where the animal became infected is situated in southern Germany, close to the Bodensee (Bodenseekreis). This area, flanked on the west by an area at high risk for TBE (Kreis Konstanz), is also at risk for TBE. From 1999 to 2006, a total of 29 autochthonous clinical cases of TBE in humans were reported in the Bodenseekreis and 35 in the Kreis Konstanz (Hellenbrand W., pers. comm.); 19% of unvaccinated forestry workers in the Bodenseekreis and 15% in the Kreis Konstanz were seropositive for TBEV (*9*). The prevalence of TBEV in ticks was 1.2%–2.3% in the Bodenseekreis (*10*).

Retrospective analyses of anamnestic data from the affected monkey park show that TBE may have appeared sporadically in macaques in the past. A monkey died in September 1995 and another in May 2006, and TBEV antigen was subsequently detected in brain tissue from the first animal and antibodies to TBEV were detected in both animals (12 U/L and 46 U/L, respectively, by ELISA). These 2 cases have not been systematically evaluated. Clinical signs of encephalitis were observed in another animal in 1999, but it seroconverted (42 U/L, ELISA test) and recovered after 2 months. Thus, TBE should be considered as a differential diagnosis in cases of encephalitis in monkeys kept outdoors in areas at risk for TBE. Further seroepidemiologic studies are planned to determine the status of TBEV infection among animals in this German monkey park. Vaccination against TBEV should be an option to protect other macaques in the group.
